# Age-Stratified Mortality Outcomes Associated with Nonselective Beta-Blocker Use in Patients with Decompensated Cirrhosis

**DOI:** 10.3390/jcm15155998

**Published:** 2026-08-01

**Authors:** Ahmad Nawaz, Avneet Kaur, Abdelkader Chaar, Ganesh Aswath, Idan Goren, Savio John

**Affiliations:** 1 Department of Gastroenterology, SUNY Upstate Medical University, Syracuse, NY 13210, USA; chaara@upstate.edu (A.C.); aswathg@upstate.edu (G.A.); goreni@upstate.edu (I.G.); johns@upstate.edu (S.J.); 2 Department of Internal Medicine, SUNY Upstate Medical University, Syracuse, NY 13210, USA; kaurav@upstate.edu

**Keywords:** cirrhosis, portal hypertension, beta-blockers, aging, mortality, acute kidney injury, real-world data

## Abstract

**Background/Objectives:** Nonselective beta blockers (NSBBs) are widely used in cirrhosis for portal hypertension, but their safety in older adults with decompensated cirrhosis remains uncertain. **Methods:** We conducted a retrospective multicenter cohort study using the TriNetX Research Network. Adults aged 30–90 years with decompensated cirrhosis were stratified into three groups: 30–60 years, 61–90 years, and a prespecified subgroup of 81–90 years. NSBB exposure (propranolol, nadolol, or carvedilol) within 30 days of decompensation was assessed, and 1:1 propensity score matching was performed within each age group. The primary outcome was 12-month all-cause mortality. Secondary outcomes included acute kidney injury (AKI), electrolyte abnormalities, and acute care utilization. **Results:** After matching, 40,074 pairs (30–60 years), 96,084 pairs (61–90 years), and 19,024 pairs (81–90 years) were analyzed. NSBB use was associated with lower 12-month mortality in ages 30–60 (4.0% vs. 4.6%; RR 0.86, 95% confidence interval [CI] 0.81–0.92). 61–90-year cohort, mortality was similar between groups (7.4% vs. 7.4%; RR 0.99, 95% CI 0.96–1.02). Patients aged 81–90 years, NSBB use was associated with higher mortality (10.9% vs. 10.0%; RR 1.08, 95% CI 1.02–1.16). Across age strata, NSBB was associated with increased AKI, hyperkalemia, and acute care visits. **Conclusions:** NSBB therapy was associated with reduced mortality in younger adults (30–60 years) but not in older patients (61–90 years) and was associated with increased mortality among those aged 81–90 years. These findings support individualized NSBB use and suggest that patient age should be considered when weighing potential benefits and risks.

## 1. Introduction

Cirrhosis is the end stage of progressive hepatic fibrosis, marked by significant architectural distortion of the liver and the formation of regenerative nodules [1]. This advanced condition reflects a decline in the liver’s synthetic functions alongside increased pressure within the portal venous system [2]. While early-stage cirrhosis may respond to targeted treatments addressing its underlying cause, potentially improving or reversing the disease, advanced cirrhosis is generally considered irreversible, leaving liver transplantation as the only definitive option. In the United States, chronic liver disease and cirrhosis represent a significant public health burden and remain among the leading causes of death [3].

Decompensated cirrhosis represents a critical progression in the disease, defined by the onset of complications directly related to cirrhosis, including ascites, hepatic encephalopathy, or variceal bleeding. The incidence of acute-on-chronic liver failure (ACLF), which is distinct from decompensated cirrhosis, varies by criteria, with 5.7 per 1000 person-years for APASL and 20.1 per 1000 person-years for EASL [4]. This progression is associated with alarmingly high mortality rates, with a 28-day mortality rate of 30%, primarily due to the limited availability of effective therapies to halt disease progression [5]. Furthermore, patients with obesity face an elevated risk of decompensation, highlighting the interplay between metabolic comorbidities and the worsening trajectory of liver disease [6].

In liver cirrhosis, portal hypertension arises from obstruction of the intrahepatic branches of the portal vein by fibrotic tissue and regenerative nodules that replace healthy liver parenchyma. This obstruction is compounded by intrahepatic vasoconstriction, driven by an imbalance between increased vasoconstrictors (e.g., endothelins, angiotensin-II, norepinephrine, thromboxane A2) and reduced endothelial vasodilators (e.g., nitric oxide) [7]. Over time, the development of portosystemic collaterals, such as gastroesophageal varices, helps divert blood flow to compensate for the elevated portal pressure [8]. As liver function declines, systemic splanchnic vasodilation occurs due to the release of vasodilating factors, further worsening portal pressure by increasing portal inflow [9]. This vasodilation also reduces central blood volume, leading to effective hypovolemia. In response, compensatory mechanisms activate, including sympathetic nervous system stimulation, renin–angiotensin–aldosterone axis activation, and increased antidiuretic hormone (ADH) production. While these mechanisms aim to maintain circulatory homeostasis, they may also be associated with severe complications such as variceal bleeding, ascites, cirrhotic cardiomyopathy, and hepatorenal syndrome [10,11].

Nonselective beta blockers (NSBBs) are the standard treatment for preventing first variceal bleeding and rebleeding, often in combination with band ligation for the latter [12]. Additionally, NSBBs have been proposed as a potential therapy to delay or prevent liver decompensation in patients with cirrhosis. The Baveno VII consensus group recommends the use of carvedilol in compensated cirrhotic patients with clinically significant portal hypertension (CSPH) to reduce the risk of decompensation [13]. Supporting this, the PREDESCI trial, a multicenter double-blind randomized controlled trial (RCT), demonstrated that NSBBs significantly lowered the incidence of clinical decompensation in patients with CSPH [14]. Over a median follow-up of 37 months, patients treated with NSBBs (either propranolol or carvedilol) experienced fewer decompensating events (17% vs. 27% in the placebo group; *p* = 0.041), with ascites being the most reduced complication. Furthermore, NSBBs decreased the likelihood of developing large varices and showed a trend toward improved survival, although this did not reach statistical significance [14].

Despite their benefits, the use of NSBBs in advanced decompensated cirrhosis warrants caution. High doses can lead to significant reductions in blood pressure and cardiac output, exacerbating circulatory dysfunction [8]. These effects are particularly concerning in patients with underlying cardiac impairment, such as cirrhotic cardiomyopathy (CCM). CCM, a common yet underrecognized complication of cirrhosis, is characterized by diastolic dysfunction, diminished systolic reserve, and electrical abnormalities, all in the absence of overt cardiovascular disease [15]. Older age and portal vein thrombosis have been identified as independent risk factors for CCM, which is associated with poorer outcomes and increased vulnerability to the adverse effects of NSBBs [16].

Given these complexities, universal use of NSBBs in patients with advanced decompensated cirrhosis may not be advisable. Instead, careful patient selection, with an assessment of cardiac and hemodynamic reserves, is essential. For patients with preserved cardiac function, low to moderate doses of NSBBs may still confer benefits while minimizing risks.

There is limited evidence on the impact of NSBBs in older adults with decompensated cirrhosis. Therefore, our study aims to fill this gap by investigating the mortality outcomes associated with NSBB use in this population, providing crucial insights into the age-dependent effects of these therapies.

## 2. Methods

### 2.1. Study Design and Data Source

We conducted a retrospective multicenter cohort study using the TriNetX Research Network, a global federated platform aggregating de-identified electronic medical records from academic and community healthcare organizations. TriNetX provides access to real-world data, including demographics, diagnoses, procedures, laboratory values, vital status, and medications mapped to standard terminologies.

### 2.2. Study Population

We identified adults aged 30 to 90 years with decompensated cirrhosis between 1 January 2015, and 31 December 2023. Decompensation was defined by the presence of ascites, variceal bleeding, spontaneous bacterial peritonitis, or hepatic encephalopathy, identified using ICD-10 codes. The index date was the earliest documented encounter reflecting hepatic decompensation.

Patients were stratified into three predefined age groups:

1. 30 to 60 years.
2. 61 to 90 years.
3. A prespecified subgroup of patients aged 81 to 90 years to assess advanced-age risk differences.

Within each age group, patients were categorized according to exposure to a nonselective beta blocker (NSBB): propranolol, nadolol, or carvedilol, prescribed within 30 days of the index date. Patients without documentation of NSBB use during this period comprised the control group.

### 2.3. Exposures and Outcomes

The primary exposure was receipt of an NSBB within 30 days of hepatic decompensation.

The primary outcome was all-cause mortality at 12 months.

Secondary outcomes included acute kidney injury, hyponatremia, hypernatremia, hypokalemia, hyperkalemia, and emergency room or urgent care visits within 12 months. Outcomes were identified through validated ICD-10 definitions and laboratory-based criteria embedded within the TriNetX platform.

### 2.4. Propensity Score Matching

To balance baseline characteristics, 1:1 nearest-neighbor propensity score matching without replacement was performed separately within each age group using a caliper of 0.1 pooled standard deviations. Covariates included age, sex, race, alcohol-associated liver disease, circulatory system diseases, neoplasms, diabetes, hypertension, chronic kidney disease, ascites, variceal bleeding, hepatic encephalopathy, and laboratory markers (sodium, creatinine, total bilirubin, albumin, international normalized ratio). Standardized mean differences of less than 0.1 were considered indicative of adequate balance.

### 2.5. Statistical Analysis

Matched cohorts were compared using risk differences, risk ratios, and odds ratios with associated 95 percent confidence intervals. Mortality was additionally assessed using Kaplan–Meier survival analysis with log-rank testing. The primary endpoint was assessed at 12 months, whereas Kaplan–Meier curves display survival over the available follow-up period. All analyses were performed using TriNetX’s built-in analytics engine. A two-sided *p*-value of less than 0.05 was considered statistically significant.

### 2.6. Ethics

The TriNetX Research Network provides access to de-identified electronic health record data. Because no identifiable patient information was accessed, this study was considered exempt from institutional review board oversight in accordance with applicable regulations.

### 2.7. AI Disclosure

No artificial intelligence tools were used in the preparation, analysis, or writing of this manuscript.

## 3. Results

### 3.1. Study Population

A total of 303,314 patients aged 30 to 90 years met criteria for decompensated cirrhosis. Of these, 47,018 (30–60 cohort), 103,952 (61–90 cohort), and 20,701 (81–90 subgroup) were prescribed an NSBB near the time of decompensation.

After 1:1 propensity score matching:

- 30–60 years: 40,074 NSBB users matched to 40,074 nonusers
- 61–90 years: 96,084 NSBB users matched to 96,084 nonusers
- 81–90 years: 19,024 NSBB users matched to 19,024 nonusers

All covariates achieved standardized mean differences below 0.1, indicating excellent balance. For patients aged 30–60 years, baseline characteristics before and after matching are presented in Table 1.

Baseline characteristics for the 61–90 year cohort are shown in Table 2.

Baseline characteristics for the 81–90 year subgroup are shown in Table 3.

### 3.2. Primary Outcome: Mortality

**Figure 1:**
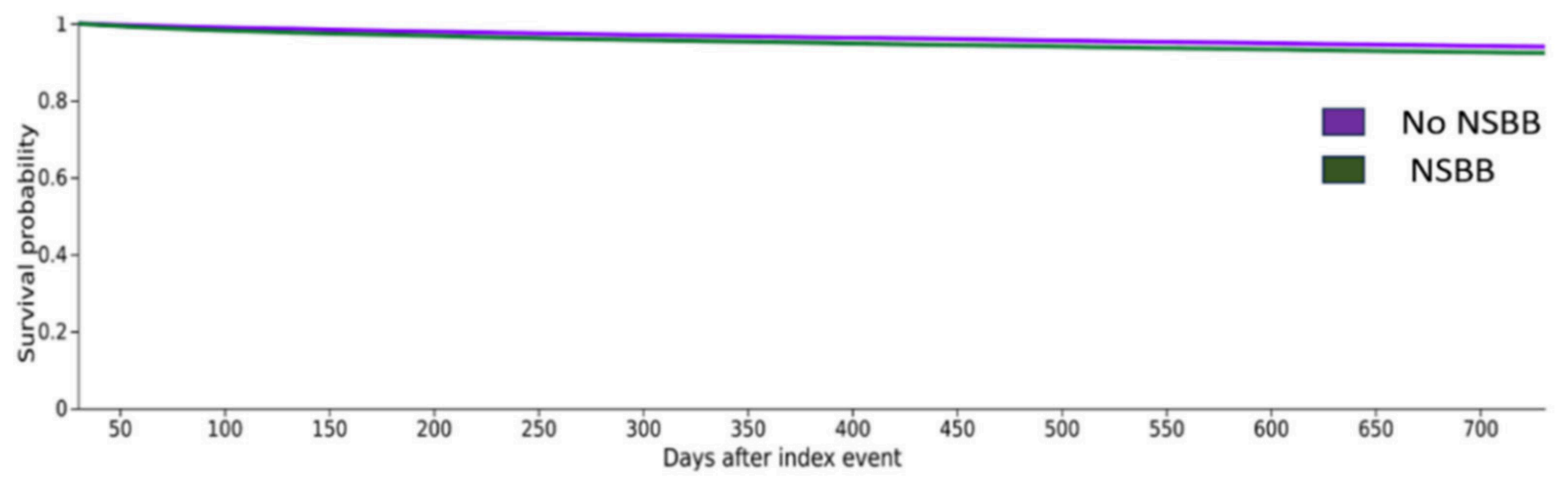
Kaplan–Meier survival curve for all-cause mortality in patients aged 30–60 years with decompensated cirrhosis stratified by nonselective beta blocker exposure.

Among patients aged 30–60 years, NSBB use was associated with lower 12-month mortality (4.0 percent vs. 4.6 percent; risk difference −0.006; risk ratio 0.86, 95 percent confidence interval 0.81 to 0.92; odds ratio 0.86, 95 percent confidence interval 0.80 to 0.92) (Figure 1).

**Figure 2:**
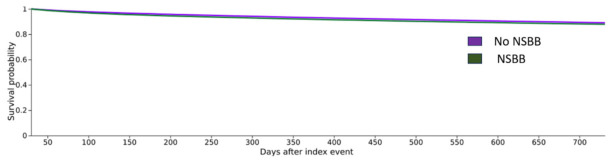
Kaplan–Meier survival curve for all-cause mortality in patients aged 61–90 years with decompensated cirrhosis stratified by nonselective beta blocker exposure.

In patients aged 61–90 years, NSBB use did not reduce mortality (7.4 percent vs. 7.4 percent; risk ratio 0.99, 95 percent confidence interval 0.96 to 1.02) (Figure 2).

**Figure 3:**
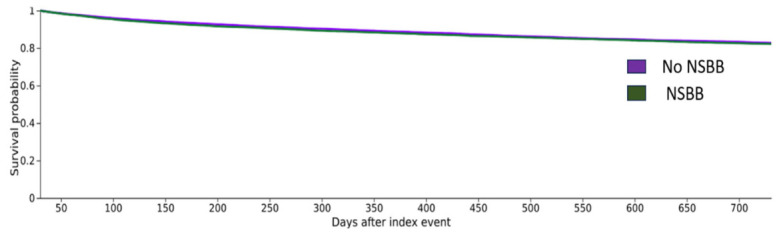
Kaplan–Meier survival curve for all-cause mortality in patients aged 81–90 years with decompensated cirrhosis stratified by nonselective beta blocker exposure.

In patients aged 81–90 years, NSBBs were associated with higher mortality (10.9 percent vs. 10.0 percent; risk difference 0.009; risk ratio 1.08, 95 percent confidence interval 1.02 to 1.16) (Figure 3).

### 3.3. Secondary Outcomes

In all age strata, NSBB use was associated with increased renal and electrolyte complications.

30 to 60 years:

- AKI: 22.6 percent vs. 18.5 percent (risk ratio 1.22, 95 percent confidence interval 1.19 to 1.26)
- Hyperkalemia: 11.9 percent vs. 8.2 percent (risk ratio 1.45, 95 percent confidence interval 1.39 to 1.51)
- ER visits: 30.7 percent vs. 26.9 percent (risk ratio 1.14, 95 percent confidence interval 1.11 to 1.16)

61 to 90 years:

- AKI: 24.3 percent vs. 18.7 percent (risk ratio 1.29, 95 percent confidence interval 1.27 to 1.31)
- Hyperkalemia: 12.9 percent vs. 8.9 percent (risk ratio 1.45, 95 percent confidence interval 1.41 to 1.49)
- Urgent care visits: 42.9 percent vs. 36.0 percent (risk ratio 1.19, 95 percent confidence interval 1.17 to 1.20)

81–90 years

- AKI: 23.8 percent vs. 19.1 percent (risk ratio 1.24, 95 percent confidence interval 1.19 to 1.30)
- Hyperkalemia: 11.3 percent vs. 8.6 percent (risk ratio 1.32, 95 percent confidence interval 1.23 to 1.41)
- ER visits: 24.6 percent vs. 20.2 percent (risk ratio 1.21, 95 percent confidence interval 1.16 to 1.26)

Across all age categories, NSBB users consistently demonstrated significantly higher risks of AKI, electrolyte abnormalities, and acute care utilization compared with nonusers.

## 4. Discussion

NSBBs are a cornerstone of portal hypertension management in cirrhosis, primarily working by reducing portal venous inflow. They achieve this through β1-blockade, which decreases heart rate and cardiac output, and β2-blockade, which induces splanchnic vasoconstriction, collectively lowering portal pressure by approximately 15% [17]. Carvedilol, an NSBB with additional α1-blocking properties, enhances intrahepatic vasodilation, making it more effective in reducing hepatic venous pressure gradient compared to propranolol or nadolol [18]. However, at higher doses, carvedilol can lower mean arterial pressure, raising concerns about hemodynamic instability [19].

Aging induces physiological and pathophysiological changes that can accelerate chronic liver disease progression and reduce treatment tolerability. Declining hepatic volume and blood flow, diminished cytochrome P450 activity, and a weakened immune response contribute to impaired liver function in older adults [20]. Beyond the liver, aging is associated with metabolic syndrome, end-organ diseases, and muscle wasting, all of which exacerbate morbidity and reduce survival in cirrhotic patients. Frailty is particularly pronounced in this population, with sarcopenia playing a key role in functional decline [21]. Older adults with cirrhosis face an increased risk of hepatocellular carcinoma, particularly those over 60 with metabolic dysfunction [22]. Autoimmune liver diseases, including autoimmune hepatitis in postmenopausal women and primary biliary cholangitis in older females, further complicate disease management [23]. Additionally, the risk of hepatobiliary malignancy in primary sclerosing cholangitis rises with age, reflecting the complex interplay between aging and liver disease progression [24]. Older adults may be more sensitive to blood pressure and heart rate-lowering effects of beta blockers, and some potential adverse effects of beta blockers such as dizziness, fatigue, cognitive impairment may be more pronounced. In addition, older adults frequently have concomitant cardiovascular disease requiring beta-blocker therapy. In such patients, use of an NSBB may reduce flexibility to prescribe or optimize cardioselective β1-blockers for competing cardiac indications. Although superiority of one beta-blocker class over another has not been consistently demonstrated for cardiovascular outcomes, NSBBs remain uniquely indicated in cirrhosis because of their effects on portal hypertension. Importantly, beta blockers are no longer considered universally contraindicated in chronic obstructive pulmonary disease, and appropriately selected agents have demonstrated favorable cardiovascular outcomes in this population. This population is also at higher risk of falls, which may be exacerbated by any blood pressure-lowering effect. Age is also an important factor in determining the suitability of therapies for portal hypertension and cirrhosis. TIPS, while effective, carries higher risks in older adults, including delayed correction of natriuresis and increased post-procedural complications [25,26]. Liver transplantation is rarely performed in patients over 70, though the number of candidates over 65 is rising [27]. Older transplant recipients face higher post-transplant mortality and increased risks of cardiac, pulmonary, and renal complications [28,29,30].

Thus, patients over 80 years of age are particularly susceptible to adverse effects owing to age-related physiological changes, polypharmacy, and a high burden of comorbidities. Given that IRBs prioritize patient safety, enrolling this age group in experimental treatments may pose an increased risk. There is also concern regarding the ability to obtain truly informed consent, as cognitive impairment is more prevalent among patients over 80 years of age and may limit their capacity to fully understand potential risks and benefits. When IRBs exclude older adults from clinical trials, it is often because alternative study designs, such as registries or observational studies, are considered safer means of generating evidence in this population. Consequently, data derived from large database-based studies may provide a valuable approach to evaluating the effects of standard, guideline-recommended therapies in this vulnerable age group.

NSBBs remain the first-line therapy for preventing variceal bleeding and have been proposed for delaying liver decompensation. However, data on their efficacy in older adults with decompensated cirrhosis remain limited. Our study found that in a propensity-matched analysis of patients aged 61–90 years, NSBB use was not associated with a significant difference in mortality [OR 0.99, 95% CI 0.95–1.02]. However, NSBB users experienced an increased risk of AKI [OR 1.39, 95% CI 1.36–1.42], hypernatremia [OR 1.24, 95% CI 1.18–1.32], hypokalemia [OR 1.11, 95% CI 1.08–1.15], hyperkalemia [OR 1.52, 95% CI 1.47–1.56], ER visits [OR 1.27, 95% CI 1.25–1.30], and urgent care visits [OR 1.33, 95% CI 1.31–1.36].

While NSBBs are generally well tolerated, their use in older adults and those with advanced decompensated cirrhosis warrants caution. Patients with cirrhotic cardiomyopathy, a condition characterized by impaired cardiac response to stress, may be particularly vulnerable, as NSBBs can further reduce cardiac output and precipitate circulatory dysfunction [31]. Additionally, high-dose NSBB therapy has been linked to increased mortality in patients with refractory ascites, likely due to worsening hemodynamics and an increased risk of hepatorenal syndrome [32,33]. In our study, subgroup analysis revealed that NSBB use in patients aged 81–90 years was associated with increased mortality compared to non-NSBB users [OR 1.10, 95% CI 1.02–1.18]. Conversely, younger patients (30–60 years) with decompensated cirrhosis demonstrated reduced mortality with NSBB use compared to nonusers [OR 0.86, 95% CI 0.80–0.92], though they also experienced increased rates of AKI, hyperkalemia, ER visits, and urgent care visits. This suggests that NSBBs may confer a modest survival benefit in younger patients, although the absolute difference in mortality was small. These findings underscore the need for a patient-specific approach, balancing the benefits of NSBB therapy with the risks of hypotension, bradycardia, renal dysfunction, and cardiac compromise in older or frail individuals.

While our study offers valuable insights, it is important to acknowledge several limitations. First, the retrospective nature of the analysis introduces potential biases, including selection bias and incomplete data capture. Second, reliance on electronic health records may result in variability in documentation quality and missing clinical details. Third, the absence of randomization limits causal inference, and residual confounding cannot be fully excluded. Additionally, modest residual imbalance in INR and serum albumin after propensity score matching may also have influenced the observed associations. Important clinical variables unavailable within the TriNetX platform included MELD-Na score, Child–Pugh class, blood pressure, heart rate, NSBB dose, dose titration or discontinuation, refractory ascites, variceal severity, endoscopic therapy, cirrhotic cardiomyopathy, frailty, and sarcopenia. These factors may have influenced both treatment allocation and outcomes despite propensity score matching. Additionally, heterogeneity in treatment protocols across institutions may affect the consistency of outcomes. Finally, the generalizability of findings may be constrained by demographic and geographic differences within the dataset.

Despite these limitations, alternative study designs or registry-based approaches are essential to gather data in older populations in a manner that is safer and more observational. In light of these factors, we believe that leveraging global real-world data platforms such as TriNetX can provide important safety signals and reveal clinically relevant trends. These findings should serve as a foundation for designing future randomized trials that are carefully structured to address the unique challenges of studying very elderly patients.

## 5. Conclusions

In conclusion, our study suggests that while NSBB therapy does not significantly affect mortality in the broader older adult cirrhotic population (61–90 years), its use in patients aged 81–90 years may be associated with increased mortality. In contrast, younger adults may derive a modest survival benefit despite experiencing higher rates of adverse events. These findings underscore the importance of individualized NSBB use in patients with decompensated cirrhosis, with careful attention to patient age, selection, dosing, and monitoring to optimize clinical outcomes. Importantly, there remains a critical need for prospective studies evaluating standard, guideline-recommended therapies in Older Adults with Liver Disease (OALD), a population that is frequently underrepresented or excluded from the clinical trials that form the evidence base for contemporary medical management.

## Figures and Tables

**Table 1:** Baseline characteristics of patients aged 30–60 years with decompensated cirrhosis receiving and not receiving nonselective beta blockers after propensity score matching.

Variable	NSBB (Matched) (*n* = 40,074)	No NSBB (Matched) (*n* = 40,074)	SMD
**Age (years), mean ± SD**	45.8 ± 8.0	45.8 ± 8.1	0.007
**White race, *n* (%)**	25,074 (62.6%)	25,179 (62.8%)	0.005
**Male sex, *n* (%)**	23,963 (59.8%)	24,086 (60.1%)	0.006
**Circulatory disease, *n* (%)**	28,143 (70.2%)	28,058 (70.0%)	0.005
**Neoplasms, *n* (%)**	9482 (23.7%)	9515 (23.7%)	0.002
**Serum sodium, mean ± SD**	136.5 ± 4.6	136.6 ± 4.5	0.023
**Serum creatinine, mean ± SD**	1.4 ± 3.5	1.2 ± 2.7	0.064
**Total bilirubin, mean ± SD**	3.4 ± 5.3	3.3 ± 6.1	0.01
**INR, mean ± SD**	1.5 ± 0.5	1.4 ± 0.6	0.15
**Serum albumin, mean ± SD**	3.2 ± 0.8	3.3 ± 0.8	0.135

Values are presented as mean ± SD or *n* (%). Propensity score matching was performed using 1:1 nearest-neighbor matching with a caliper of 0.1 pooled standard deviations.

**Table 2:** Baseline characteristics of patients aged 61–90 years with decompensated cirrhosis receiving and not receiving nonselective beta blockers after propensity score matching.

Variable	NSBB (*n* = 96,084)	No NSBB (*n* = 96,084)	SMD
**Age (years), mean ± SD**	65.5 ± 8.5	65.5 ± 8.6	0.006
**White race, *n* (%)**	60,904 (63.4%)	61,392 (63.9%)	0.011
**Male sex, *n* (%)**	56,597 (58.9%)	56,473 (58.8%)	0.003
**Circulatory disease, *n* (%)**	74,555 (77.6%)	74,455 (77.5%)	0.002
**Neoplasms, *n* (%)**	39,694 (41.3%)	40,103 (41.7%)	0.009
**Serum sodium, mean ± SD**	137.3 ± 4.5	137.1 ± 4.4	0.03
**Serum creatinine, mean ± SD**	1.4 ± 3.2	1.3 ± 3.0	0.042
**Total bilirubin, mean ± SD**	2.0 ± 3.3	2.0 ± 3.8	0.003
**INR, mean ± SD**	1.4 ± 0.5	1.3 ± 0.6	0.056
**Serum albumin, mean ± SD**	3.2 ± 0.7	3.3 ± 0.8	0.072

Values are presented as mean ± SD or *n* (%). Propensity score matching was performed using 1:1 nearest-neighbor matching with a caliper of 0.1 pooled standard deviations.

**Table 3:** Baseline characteristics of propensity score–matched patients aged 81–90 years with decompensated cirrhosis receiving and not receiving nonselective beta blockers.

Variable	NSBB (*n* = 19,024)	No NSBB (*n* = 19,024)	SMD
**Age (years), mean ± SD**	84.9 ± 2.8	84.9 ± 2.8	0.004
**White race, *n* (%)**	13,130 (69.0%)	13,146 (69.1%)	0.001
**Male sex, *n* (%)**	9554 (50.2%)	9571 (50.3%)	0.002
**Circulatory disease, *n* (%)**	14,155 (74.4%)	14,130 (74.3%)	0.002
**Neoplasms, *n* (%)**	8615 (45.3%)	8633 (45.4%)	0.002
**Serum sodium, mean ± SD**	137.1 ± 4.6	137.0 ± 4.6	0.015
**Serum creatinine, mean ± SD**	1.2 ± 2.3	1.1 ± 2.2	0.041
**Total bilirubin, mean ± SD**	1.7 ± 2.9	1.7 ± 3.2	0.002
**INR, mean ± SD**	1.3 ± 0.4	1.3 ± 0.5	0.008
**Serum albumin, mean ± SD**	3.3 ± 0.7	3.3 ± 0.8	0.017

Values are presented as mean ± SD or *n* (%). Propensity score matching was performed using 1:1 nearest-neighbor matching with a caliper of 0.1 pooled standard deviations.

## Data Availability

The data supporting the findings of this study are available through the TriNetX Research Network to participating institutions. Restrictions apply to the availability of these data due to licensing agreements with TriNetX.
